# Analyses of crystal growth, optical, electrical, thermal and mechanical properties of an excellent detector-grade Cd_0.9_Mn_0.1_Te: V crystal

**DOI:** 10.1038/s41598-020-59612-0

**Published:** 2020-02-17

**Authors:** Lijun Luan, Li Gao, Haohao Lv, Pengfei Yu, Tao Wang, Yi He, Dan Zheng

**Affiliations:** 1 School of Materials Science and Engineering, Chang’an University, Xi’an, Shaanxi 710064 China; 2 School of Materials Science and Engineering, Northwestern Polytechnical University, Xi’an, Shanxi 710072 China

**Keywords:** Electronic devices, Characterization and analytical techniques

## Abstract

A high-quality cadmium manganese tellurium (Cd_0.9_Mn_0.1_Te: V or VCMT) crystal was successfully grown via modified Te solution vertical Bridgman method with vanadium doping. The crystal structure and quality were evaluated by powder X-ray diffraction analysis. An infrared transmission spectroscope measured the transmittance of the crystal at 64%, which would suggest that the grown crystal possessed high purity and crystallinity. Ultraviolet-visible-near-infrared spectroscopy analysis obtained the forbidden band width of approximately 1.577 eV. The current-voltage test indicated that the VCMT crystal had a high resistivity of 2.07 × 10^10^ Ω·cm. Mechanical properties were measured by a Vickers microhardness tester. Crack surface morphology around the indentation was recorded. Furthermore, mechanical properties, such as microhardness, fracture toughness, brittleness index and yield strength were investigated and discussed. The thermal stability of the VCMT single crystal was determined by thermogravimetric analysis. A VCMT detector was fabricated with planar configuration structure, which showed a resolution of 11.62% of the ^241^Am at 59.5 keV peak.

## Introduction

In the late 1970s, Polish scientist Galazka^1^ introduced the excellent properties of cadmium manganese telluride (Cd_1−x_Mn_x_Te) crystals. As a typical diluted magnetic semiconductor,Cd_1−x_Mn_x_Te has been widely studied. Seo *et al*.^2^ found that this semiconductor can provide different coordination fields by doping with chromium (Cr), thereby making Cr-doped Cd_1−x_Mn_x_Te a tunable mid-infrared laser material with a wavelength range of 2.17–3.01 μm. Brost *et al*.^3^ found that Cd_1−x_Mn_x_Te possesses strong photorefractive properties in the near-infrared region and can be applied to optical communication. Roy *et al*.^4^ also proposed that adding selenium (Se) was highly effective in reducing secondary phases (Te inclusions) and lattice hardening, thereby resulting in CdZnTe free from sub-grain boundary networks. From the point of view of material performance, Cd_1−x_Mn_x_Te has potential advantages over CdZnTe in X-ray and gamma ray detector applications. Cd_1−x_Mn_x_Te has been reported as a highly promising material for fabricating X-ray and gamma ray detectors at room temperature^5–8^.

As theCd_1−x_Mn_x_Te crystal has excellent application prospects, cultivating a single crystal with good quality is necessary, as well as studying its crystalline perfection and mechanical and thermal properties, to lay a solid foundation for future applications. However, the mechanical properties of semiconductor materials change due to dislocations introduced by the fabrication process of devices. These dislocations affect carrier density, carrier mobility and lifetime, thereby influencing the photoelectric properties of the crystal^9^. Numerous studies have been conducted on Cd_1−x_Mn_x_Te crystal, particularly on the correlations between crystal growth, quality evaluation and properties. However, to date, limited research has been published on the mechanical properties of the Cd_1−x_Mn_x_Te crystal. Therefore, understanding the mechanical properties for controlling the crystal growth is important, as well as eliminating the dislocations and improving the photoelectric property of the crystals. The vertical Bridgman (VB) method is widely used to grow Cd_1−x_Mn_x_Te crystals. Roy *et al*.^10^ proposed that the VB method can grow Cd_1−x_Mn_x_Te crystals without large Te inclusions. However, for the crystals grown through this method, the high tendency of twin formation is a severe problem in device application. The Te-rich solution method can be used to grow a Cd_1−x_Mn_x_Te single crystal below the phase-transition temperature to reduce the occurrence of phase-transition twins^11^. Moreover, using vanadium as a doping impurity can compensate for double-ionised Cd vacancies and shallow level impurities to increase the resistivity of the Cd_1−x_Mn_x_Te crystal^12^.

In this work, Cd_0.9_Mn_0.1_Te: V (VCMT) single crystal with vanadium doping concentration of 5 × 10^17^ atoms/cm^3^ was grown by Te solution (10% excess) VB method. The quality evaluation and crystal structure were analysed by powder X-ray diffraction (XRD), ultraviolet-visible-near infrared (UV-VIS-NIR) spectra and Fourier transform infrared spectroscope (FT-IR) to provide information on the material band gap width. The mechanical strength of the crystal was determined by a Vickers indentation tester, and the mechanical properties of microhardness (*H*_v_), fracture toughness (*K*_c_), brittleness index (*B*_i_) and yield strength (*δ*_y_) were systematically studied. Then, the thermal stability of the crystal was examined by thermogravimetric analysis (TGA). The current-voltage (*I*-*V*) characteristics and gamma-ray spectral were measured to calculate the resistivity and the energy resolution of the crystalfor the detector application.

## Results and Discussion

### Crystal structure and quality analysis

**Figure 1:**
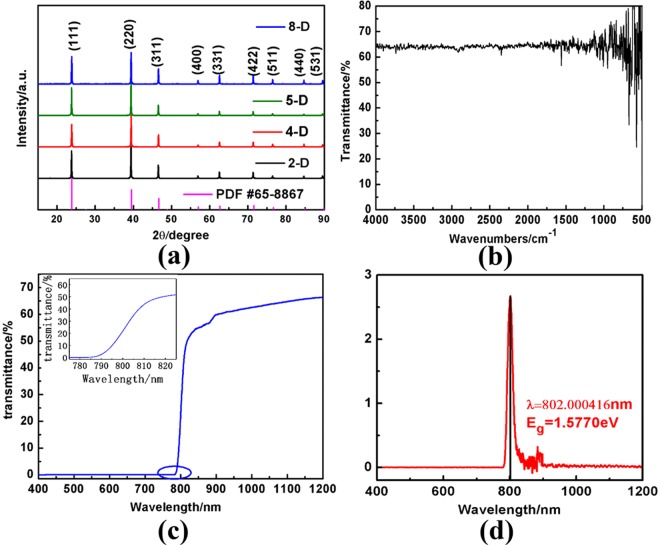
(**a**) XRD patterns of as-grown VCMT crystals and standard card with No. 65-8867, (**b**) typical IR transmittance spectrum of sample 5-D, (**c**) typical UV-VIS-NIR spectrum, and (**d**) derivative curve of UV-VIS-NIR spectrum.

The crystal structure and quality analysis of the VCMT crystal were investigated by X-ray powder diffraction method. The XRD patterns of as-grown crystal and the standard patterns (PDF No. 65-8867) are shown in Fig. 1(a), where all the results indicate that the crystal is characterised by a zinc-blende structure, which implies that the crystal growth process successfully restrains the solid phase transformation. The diffraction patterns also show that the diffraction spectra of various samples are strikingly similar. However, the positions of the diffraction peaks corresponding to the same lattice planes in different samples deviated from each other when certain peak positions were zoomed in. The reason is that the Mn content varies slightly in different axial positions in the ingot although the segregation coefficient of Mn in CdTe is close to 1^10^. The data on statistical full width at half-maximum (FWHM) are shown in Table 1, where the FWHM value of sample 5-D is the smallest, thereby indicating that the crystal quality of sample 5-D is the best. The lattice constant of the VCMT crystal can be accurately calculated using the following extrapolation function proposed by Nelson:

1 $$a=\frac{co{s}^{2}\theta }{2}(\frac{1}{sin\theta }+\frac{1}{\theta }).$$

**Table 1:** FWHM data of different diffraction planes (unit: °).

	Diffraction crystal planes								
Wafers	(111)	(220)	(311)	(400)	(331)	(422)	(511)	(440)	(531)
2-D	0.142	0.126	0.142	0.168	0.138	0.163	0.172	0.157	0.204
4-D	0.146	0.123	0.142	0.180	0.151	0.163	0.169	0.141	0.209
5-D	0.121	0.094	0.122	0.140	0.116	0.141	0.156	0.164	0.174
8-D	0.146	0.132	0.143	0.137	0.125	0.138	0.139	0.142	0.167

According to the powder XRD data and Eq. (1), the calculated lattice constant is 6.4684 Å. This result is well matched with that of undoped VCMT crystal, thereby showing that the vanadium doping does not change the crystal structure and lattice constant.

### FT-IR spectrum analysis

FT-IR is a simple and effective non-destructive testing method and an important way to characterise the quality of VCMT crystals. The transmission spectrum of a middle-position sample with good quality was measured by using a Nicolet Nexus FT-IR spectrometer (Thermo Fisher Scientific, US) in a wave number range of (4000–500) cm^−1^. At room temperature, the testing results are shown in Fig. 1(b). The figure indicates that the IR transmittance of the middle-position sample is up to 64%, which is slightly lower than the theoretical transmittance of 65%^13^. Du Yuanyuan *et al*.^14^ reported that the IR absorption behaviour of the VCMT crystal was mainly affected by the lattice absorption and free carrier absorption. As CdZnTe has been fully developed in the interpretation of the infrared transmittance, the same method can be applied to VCMT. The absorption of IR radiation by crystals at room temperature is mainly related to the scattering of Te precipitation/inclusion phase and free carrier absorption. The corresponding transmittance (T4000) at the wave number of approximately 4000 cm^−1^ is dominated by the scattering of Te precipitation/inclusion phase, andT500 is dominated by the absorption of free carrier. In Fig. 1(b), the infrared transmission curve of the wafer shows a minor fluctuation and the infrared transmittance is basically unchanged from 4000 cm^−1^ to 500 cm^−1^, thereby indicating that the crystal quality and homogeneity are satisfactory.

### UV-VIS-NIR spectrum analysis

VCMT crystal is classified as a direct bandgap semiconductor. In addition, when the photon energy is greater than the bandgap width, the electrons jump from valence band to conduction band, and then generate electron-hole pairs. This process is called intrinsic absorption. According to the absorption law of inter-band transition of semiconductor materials, absorption coefficient dot photon energy is proportional to half of the power of the difference of photon energy and direct bandgap width, that is,

2 $$\alpha \cdot hv=B{(hv-{E}_{g})}^{1/2},$$

where *α* is the absorption coefficient of the semiconductor VCMT, *B* is a constant, *hv* is the photon energy and *E*_g_ is the bandgap width of the semiconductor. Therefore, the bandgap width of the direct band gap semiconductor can be obtained.

In this experiment, the VCMT sample grown by the Te solution VB method was used. The band gap width could be determined by the Mn content x = 0.1. The room-temperature UV-VIS-NIR spectrum of the VCMT indicated that the absorption edge of VCMT was approximately 800 nm, as shown in Fig. 1(c). As an approximately vertical line, the absorption edge divides the spectrum into two parts: when the wavelength is less than 785 nm, that is, the incident photon energy is greater than the bandgap width, the electrons in the valence band absorb all of the photon energy and jump into the conduction band, thereby inducing zero transmittance, which is the strong absorption region. When the incident wavelength is within the range of (785–815) nm, as shown in the inset of Fig. 1(c), the absorption coefficient presents an exponential change rule, which is an exponential absorption region. When the incident wavelength is greater than 850 nm, the absorption coefficient is extremely small, which is a weak absorption region^15^. To confirm the cutoff wavelength, we obtained the derivative curve of the spectrum in Fig. 1(c). This spectrum is presented in Fig. 1(d), where the peak value of the derivative curve is the corresponding point of the cutoff wavelength. Based on this value, the cutoff wavelength λ was approximately equal to 802 nm and the bandgap width *E*_g_ was equal to 1.577 eV. The fitting results indicated that the vanadium doping did not significantly induce the band gap variation of the VCMT crystal.

### Electrical property

**Figure 2:**
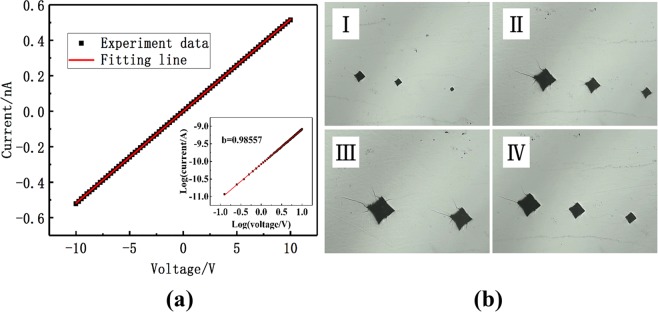
(**a**) Typical *I-V* curve, and (**b**) typical indention marks and cracks on a (111) face of VCMT crystal.

The current-voltage (*I*-*V*) test is a non-destructive common method for analysing the resistivity of semiconductor materials. Figure 2(a) shows the linear *I*-*V* characteristic curve at the positive and negative bias voltage, as a result of it indicating an ohmic contact between Au electrodes and the VCMT wafer. The ohmic characteristics can be quantitatively analysed on the basis of the linear exponent of the *I*-*V* curve. The *I*-*V* results can be fitted according to the following equation^16^:

3 $$I=a{V}^{b},$$

where *a* is a constant and *b* is an ohmic characteristic coefficient. For an ideal ohmic contact, *b* should be exactly equal to 1. When *b* is close to 1, the result is close to the ohmic contact. Then, fitting result *b* = 0.98557 is shown in the inset in Fig. 2(a), indicating an approximate ohmic contact between the crystal and surface electrodes.

Then, we calculate the resistivity of the crystal using Ohm’s law^17^, which is expressed as

4 $$\rho =\frac{RS}{L}=\frac{VS}{IL},$$

where *R* is the bulk resistance of the VCMT crystal, *V* is the test voltage, *I* is the test current at voltage *V*, *S* is the contact area of the electrodes and wafer and *L* is the thickness of the wafer. In Fig. 2(a), the fitting curve is indicated by the thin, solid line. The resistivity of the VCMT crystal is calculated to be 2.07 × 10^10^ Ω·cm, which shows that the crystal quality is satisfactory. The reasons for the successful cultivation of high-resistivity VCMT crystal in the excess Te solution are the following: first, sufficient Te_Cd_ can compensate for the native point defect V_Cd_ in the VCMT crystal, and second, the shallow donor impurity vanadium can effectively compensate for the Cd vacancy. These two aspects reduced the free carrier density and increased the resistivity.

### Microhardness analysis

The mechanical properties of the crystals play an essential role in their physical stability and device fabrication^18^. In this study, the mechanical properties of VCMT crystals were analysed through a microhardness tester at room temperature. During the test under different loads, the indentation sizes and crack surface morphology were photographed by a microscope equipped with the hardness tester and shown in Fig. 2(b).

**Figure 3:**
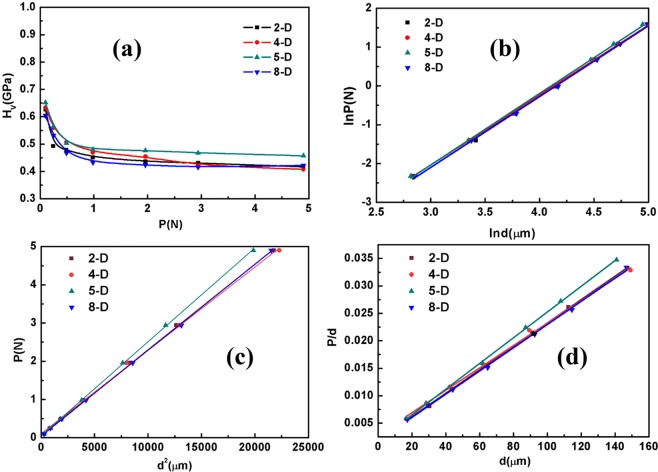
(**a**) Load (*P*)-dependent microhardness (*H*_*V*_) graph, (**b**) ln*P* vs. ln*d* plots, (**c**) plots of *P* vs. *d*^*2*^, (**d**) andplots of *P/d* vs. *d*.

Under diverse loading conditions (*P*), the indentation sizes (*d*) were directly derived from the CCD image processing software. The hardness value *H*_v_ was obtained as follows:

5 $${H}_{V}=1.854P/{d}^{2},$$

where 1.854 is the geometric conversion factor, *P* is the applied load and *d* is the indenter dimension. The load-dependent Vickers hardness number *H*_v_ is indicated in Fig. 3(a). As shown in this graph, with the increased load, the indentation size also increased but the microhardness number *H*_v_ decreased. This tendency indicates that the crystal follows the normal indentation size effect (ISE). As shown in Fig. 3(a), the wafer 5-D possesses a higher hardness than that of other wafers; this condition is correlated with the crystalline perfection of the crystal discussed in the section on ‘Crystal structure and quality analysis’. The measured values of the load-independent hardness number *H*_*V*_ were given in Table 2, and were somewhat higher than the hardness value 0.59 GPa of Cd_1−x_Mn_x_Tegrown by the vertical Bridgman method reported by Hwang *et al*.^9^.

**Table 2:** Calculated mechanical parameters.

Samples	Load independence hardness value *H*_V_(GPa)	Meyer index number(*n*)	Hays–Kendall approach		PSR model		Fracture toughness *K*c(GP*a∙*m^1/2^)	Brittleness index *B*_i_ (m^−1/2^)	Yield strength *δ*_*y*_(GPa)
			*W* (N)	*A*_*1*_	*a*	*b*			
2-D	0.62	1.83	0.063	0.000224	0.0019	0.000213	0.0020	218.4	0.209
4-D	0.63	1.80	0.052	0.000246	0.0018	0.000204	0.0016	214.1	0.211
5-D	0.65	1.84	0.104	0.000218	0.0028	0.000234	0.0021	303.2	0.217
8-D	0.60	1.82	0.037	0.000225	0.0018	0.000211	0.0013	337.7	0.201

The simplest way to explain the ISE is Meyer’s law, which provides the formula on load and indentation size as follows^19^:

6 $$P=A{d}^{n},$$

where *P* is the applied load, *d* is the diagonal length of the indentation, *A* is a constant for a given material and *n* is the Meyer’s index, namely, the work hardening coefficient. For normal ISE behaviour, the index (*n*) should be less than 2. When *n* is equal to 2, the hardness is independent of the applied load and is given by Kick’s law^19^ as follows:

7 $$P={A}_{0}{d}^{2},$$

where *A*_0_ is the geometric conversion factor of the indenter, and *P* and *d* represent the same meanings as those in Eq. (6). The relationship between *lnP* and *lnd* is shown in Fig. 3(b), where every plot of *lnP* versus *lnd* shows a linear relationship in which the slope represents the value of *n*. The best fitting data for *n* are given in Table 2. The table shows that the Meyer’s index of every sample expresses the value *n* < 2, thereby indicating that these samples exhibit normal ISE behaviour^20,21^. Onitsch and Hanneman pointed out that if the value of *n* falls between 1 and 1.6, then the material is hard, and if *n* > 1.6, then the material is soft. The value of *n* for the VCMT crystal is approximately 1.8, which suggests that the grown crystal is slightly soft.

Hays and Kendall proposed that elastic deformation always occurs at a load range of no more than *W*. According to Hays–Kendall’s^22^ approach, the load dependence of the hardness is given by

8 $$P=W+{A}_{1}{d}^{2},$$

where *W* is the largest load that corresponds to elastic deformation and *A*_1_ is the load-independent constant, which provides the hardness value independent of the load. *P* and *d*^2^ are well matched with a linear relationship in the entire range of *d*, as shown in Fig. 3(c), where the intercept of the vertical axis is *W* and the slope of the straight line is *A*_1_; both parameters are reported in Table 2. The literature^20,23,24^ showed that the positive value of *W* represents the normal ISE. Thus, in the present indentation analysis, all the wafers show positive values (*W*), which suggest the occurrence of ISE behaviour. According to this method, the larger the *W*, the larger the elastic deformation. The largest load *W* we obtained is no more than 0.104N, which suggests that our crystal has a low level of elastic deformation.

The proportional sample resistance (PSR) model is suitable for analysing the load dependence (elastic deformation range) and load independence (plastic deformation range) of the hardness. According to the PSR model^22^, the relationship between the indentation load (*P*) and indentation size (*d*) is expressed as follows:

9 $$P=ad+b{d}^{2},$$

where *a* and *b* are constants for the given material and are respectively related to the elastic and plastic properties of the material, *a* characterises the relationship between hardness and load and *b* is a constant independent of the load. As shown in Fig. 3(d), according to the plots of *P/d* versus *d* of the samples, the values of the constants *a* and *b* are calculated and listed in Table 2. According to this method, the positive values of *a* and *b* represent normal ISE. That is, the *H*_V_ value decreases as the loading effect increases^19^. The experimental results of low *ad* and low *bd*^2^ correspond to low surface energy and low plastic deformation in this material, and the positive values of *a* and *b* indicate the normal ISE behavior and applicability of the model^20^.

**Figure 4:**
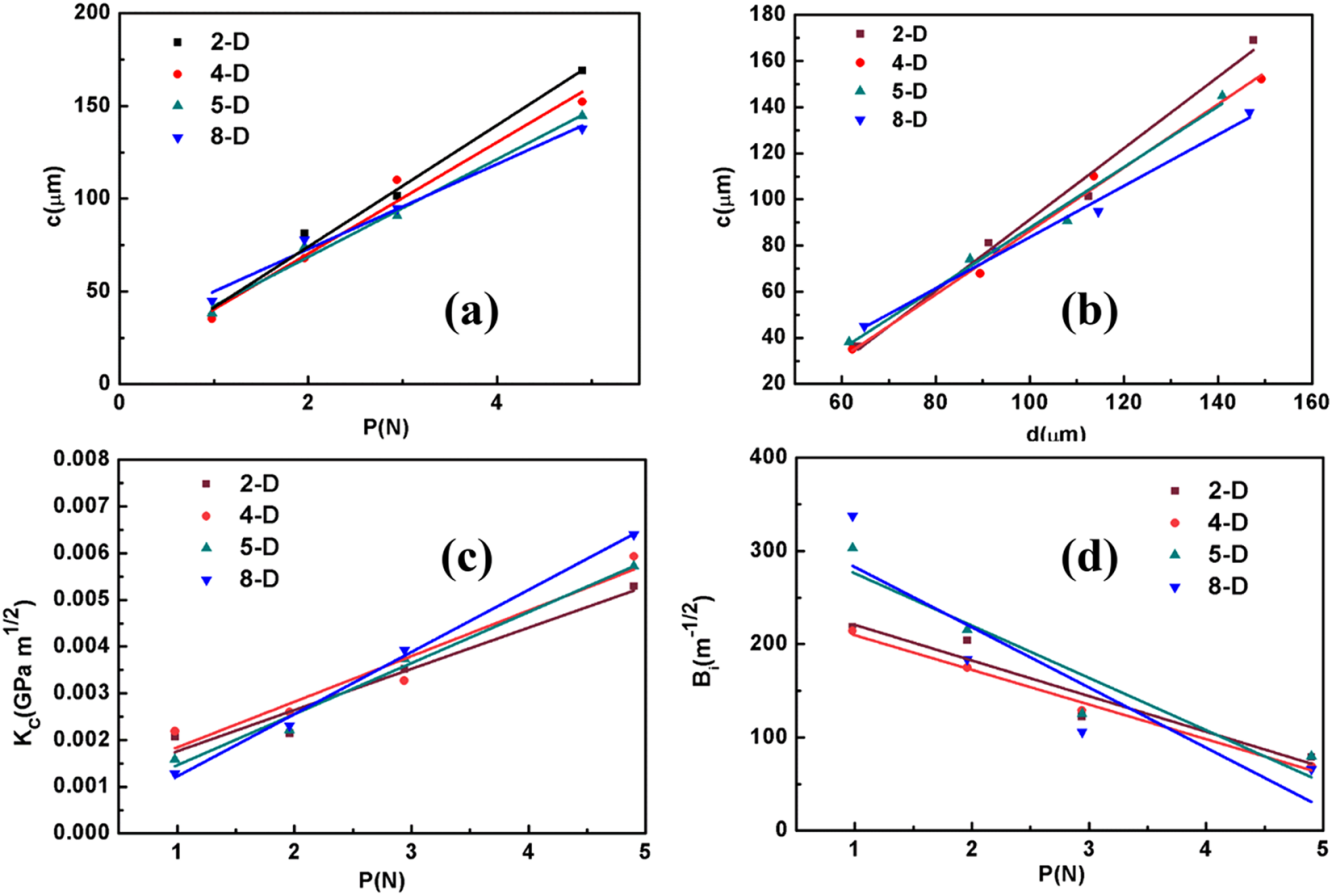
(**a**) Load *P*-dependent of crack length *c*, (**b**) relationship between crack length *c* and indenter diagonal length *d*, (**c**) plots of various fracture toughness *K*_C_ with load *P*, and (**d**) brittleness index *B*_i_ with load *P*.

Fracture toughness reflects the toughness of the material and shows how much of the fracture is induced under constant loading. When the load exceeds the limit or yield point, it becomes a key parameter in selecting the applied materials^25^. According to Ponton and Rowlings^26^, two types of crack systems can develop in a material as a result of compression: (i) central radial cracks and (ii) Palmqvist crack systems. The crack length (c) was measured from the centre of the indentation to the tip of the crack. When *c*/*a* ≥ 2*.5* (where *a* = *d*/*2*), the crack generated around the indentation belongs to a median crack. If *c*/*a* ≤ *2.5*, then the crack formed around the indentation is called Palmqvist crack. In the current study, the significant crack appeared in the corner of the indentation with *p* = 0.98N and *c/a* ≤ *2.5*, thereby suggesting that the crack system in our sample is a Palmqvist crack. That the Palmqvist crack is formed at a load of 0.98N means that the grown VCMT crystal has a relatively high mechanical strength. As shown in Fig. 4(a,b), the crack length is linear with the applied load. The fracture toughness of the material can be calculated through the following relationship^26^:

10 $${K}_{C}=\frac{Pa}{{\beta }_{0}{l}^{1/2}},$$

where *K*_*C*_ is the fracture toughness (GPa∙m^1/2^), *c* is the radial crack length (μm), *β*_0_ = 1/7 is a constant and *l* = *c* - *a* is the mean Palmqvist crack length. The values of *K*_*C*_ estimated by Eq. (10) at each load value are shown in Fig. 4(c). The obtained *K*_*C*_ values are in the range of (0.0013–0.0021) GPa∙m^1/2^, which is presented in Table 2. The strong dependence of *K*_*C*_ on the surface crack length indicates that *K*_*C*_ has a characteristic of residual surface stress. The change in crack length and fracture toughness with the loading can be attributed to the deep penetration of the indenter against the surface^27^.

Brittleness (*B*_*i*_) is an important property of crystals, the index of which determines the fracture strength of crystals without obvious deformation. This property is expressed by the following equation^28^:

11 $${B}_{i}={H}_{V}/{K}_{C},$$

where *H*_*V*_ is the Vickers hardness value and *Kc* is the fracture toughness. The variations in the values of *B*_*i*_ at each load are shown in Fig. 4(d) and the calculated *B*_*i*_ values in the range of (214.1–337.7) *m*^1/2^ are presented in Table 2.

For a material of a Meyer’s index *n* < *2*, the yield strength (*δ*_*y*_) can be calculated using the following formula^23^:

12 $${\delta }_{y}={H}_{v}/3,$$

where *δ*_*y*_ is the yield strength and *H*_*V*_ is the microhardness of materials. The calculated yield strength value, which I s given in Table 2, is slightly more than 0.2 GPa.

### Thermal analysis

**Figure 5:**
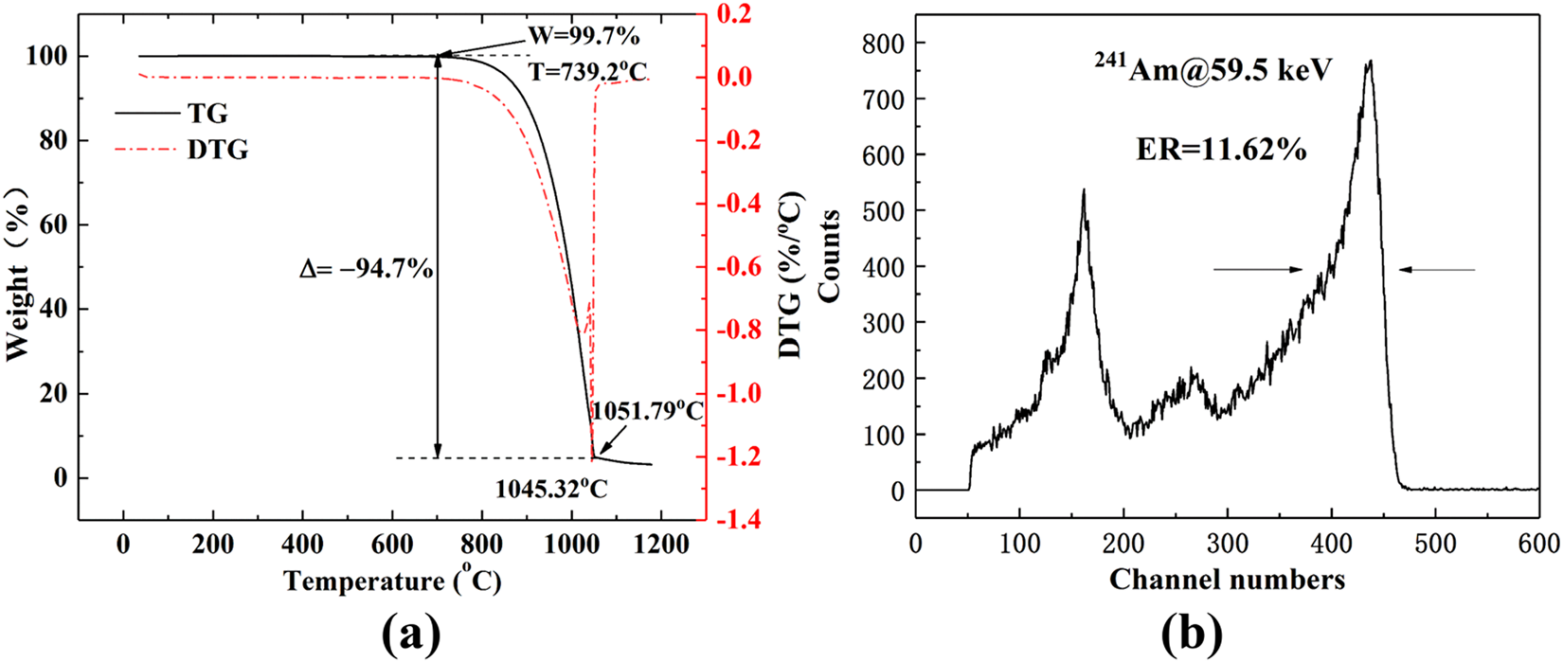
(**a**) TG/DTG curves, (**b**) atypical ^241^Am at 59.5 keV gamma-rayspectroscopyresponsefrom VCMT detector.

The thermal stability of VCMT was determined by using a thermogravimetric analyser (TGA). The sample was heated to 1200 °C in a nitrogen atmosphere, and the results obtained from TGA are shown in Fig. 5(a). The weight loss occurred in the temperature range of 739.2–1051.79 °C, where most of the compounds were decomposed into gaseous products. The beginning reference temperature point was 739.2 °C, which marked the beginning of the weight loss process of the VCMT sample, thereby indicating that the VCMT crystal had high thermal stability. The end reference temperature point of the weight loss process is 1051.79 °C. The decomposition rate reached a maximum value (DTG peak) at 1045.32 °C.

### Gamma-ray spectral measurements

The energy response spectroscopy of VCMT detector irradiated by ^241^Am at 59.5 keV as a function of bias voltage at room temperature was acquired using a standard Multi-Channel Analyzer (MCA) system. A typical ^241^Am at 59.5 keV gamma-ray spectroscopy response from VCMT detector is shown in Fig. 5(b). The bias voltage is 200 V, and the shaping time is 2 μs. It can be seen that the energy spectrum is clear and the position of the omnipotent peak is obvious, indicating that the crystal quality has completely reached the detector level. The measured energy resolution of 59.5 keV is about 11.62%, which is totally compared with the works of Du *et al*.^14^ for the CMT detector at 59.5 keV of 12.51% resolution and Yu *et al*.^29^ for the annealed CMT:In detector of 12.32% resolution. The reason why the grown VCMT crystal fully meets the requirements of making radiation detector at room temperature is that the Te solution Bridgman method can lower the growth temperature, avoid crucible impurity pollution, and reduce intrinsic defects. In addition, V doping can effectively compensate the Cd vacancy generated during crystal growth and recombine the shallow level defects in the crystal.

## Conclusions

The successful growth of a large single crystal of vanadium-doped Cd_0.9_Mn_0.1_Te: V was achieved by the VB technique. The crystal was characterised in terms of optics, electricity, mechanics and thermology. The perfect crystallinity and zinc-blende structure of the growth crystal were proved by the powder XRD data. 5-D wafer was confirmed to have the best crystalline perfection because of the lowest value of FWHM. The IR transmittance of the central wafer of the crystal was up to 64%, which was close to the theoretical transmittance. The VCMT crystal exhibited a cutoff wavelength of 802 nm with a band gap of 1.577 eV. The I-V test indicated that the VCMT crystal had a high resistivity of 2.07 × 10^10^ Ω∙cm. The mechanical and thermal properties of the VCMT crystal were systemically investigated for the first time. Classical Meyer’s law, Hays–Kendall’s law and the PSR model were applied to analyse the microhardness behaviour. Important mechanical parameters of microhardness, fracture toughness and yield strength were obtained corresponding to the values of (0.6–0.65) GPa, (0.0013–0.0021) GPa∙m^1/2^ and (0.201–0.217) GPa, respectively. The excellent mechanical properties of 5-D wafer were effectively correlated with the XRD research results. Thermal analysis showed that the VCMT possessed high thermal stability. Moreover, the energy resolution at 59.5 keV peak is 11.62%, demonstrating that the as-grown VCMT crystal has met the demands of requirement for the nuclear detector applications.

## Methods

### Crystal growth

Vanadium-doped Cd_0.9_Mn_0.1_Te (VCMT) ingots were grown by the Te solution VB method, which can reduce the twins of VCMT crystals more effectively than the primary VB method. The high-purity raw materials of Cd (7N), Mn (5N) and Te (7N) doped with vanadium (4N) at a concentration of 1 × 10^17^ atoms/cm^3^ were loaded into a carbon-coated quartz crucible (6N) and sealed under vacuum of 1 × 10^−4^ Pa. In addition, approximately 10 at% of Te was used as a solvent to reduce the melting point of the crystal and avoid the phase transformation. Then, the sealed crucible was placed in a self-designed rocking furnace to synthesise multicrystalline VCMT. Multicrystal synthesis and single growth were conducted in the same crucible to reduce the introduction of the impurities during the operation. The crucible was placed in a two-zone furnace to grow single crystals. The growth rate was 0.1 mm/h and the temperature gradient around the melt/solid interface was (10–15) K/cm. After growth, the ingot was cooled to room temperature at a rate of 20 K/h. Then, the grown ingot was removed from the crucible coated with carbon film, thereby ensuring that the ingot could be easily taken out of the crucible.

**Figure 6:**
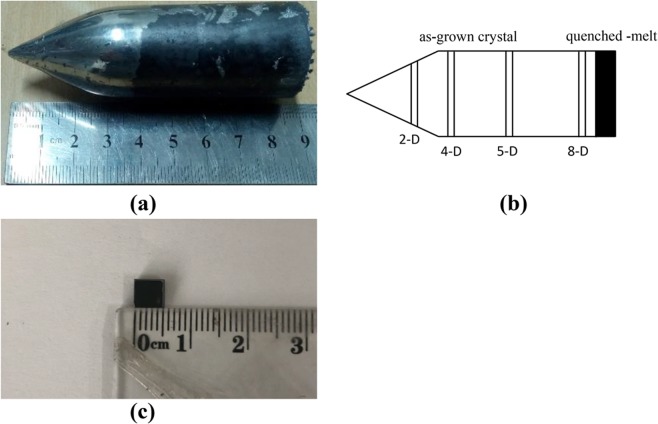
(**a**) grown VCMT ingot with 30 mm diameter and 90 mm length, (**b**) a schematic view of the cutting position of the wafer, and (**c**) individual wafer cut from ingot with dimensions of 5 × 5 × 2 mm^3^.

Finally, the VCMT ingot with a diameter of 30 mm and length of 90 mm was obtained, as shown in Fig. 6(a). Anda schematic view of the cutting position of the wafer is shown in Fig. 6(b). Then, the as-grown VCMT ingot was cut into individual wafers with a dimension of 5 × 5 × 2 mm^3^ by a linear cutting machine that cut perpendicular to the growth direction. As shown in Fig. 6(c), the as-cut wafers were mechanically polished on two sides using MgO powders and silica solution in turn as grinding media to obtain a mirror-like surface. Then, the wafers were chemically polished with 2% bromine–methanol solution for 2 min to remove the mechanical damage layer on the surface.

### Measurements

To evaluate the crystal structure and crystallinity of the as-grown VCMT crystal, powder XRD analysis was conducted by an AXS D8 Advance diffractometer equipped with Cu Kα radiation. The diffraction peaks were recorded in the 2θ range of 15°–90° with a scan step of 0.02°. The IR transmittance measurements were performed at room temperature using a Nicolet Nexus FT-IR spectrometer with wave number in the range of (4000–500) cm^−1^. The UV-VIS-NIR transmittance spectra of the crystals were recorded using a Shimadzu UV-3150 spectrophotometer in the range of (400–1200) nm. Microhardness is important for the mechanical properties of materials. The Vickers microhardness of the as-grown crystals was tested by an HV-1000A microhardness tester (Huayin Co. Ltd., China) equipped with a diamond square indenter at room temperature. The loading range was 0.1–5.0N with a constant indentation time of 15 sec. Various mechanical parameters, such as *H*_v_, *K*_*c*_, *B*_i_ and *δ*_y_, were calculated. Thermal studies were conducted using a thermal analysis instrument, which was an SDT 650 simultaneous thermogravimetric analyser. The temperature scanning range was up to 1200 °C at a rate of 20 k/min in the nitrogen atmosphere. After being polished, the samples were plated with the Au electrodes on both sides in the symmetrical positions of the samples by vacuum evaporation. Then, the resistivity of the sample was tested using Keithley 6517A semiconductor parameter test equipment. In addition, the exposed VCMT surface was passivated with 30% H_2_O_2_ solution for several minutes to reduce the surface leakage current. The energy resolution of the detector was measured with a standard ORTEC measurement system at room temperature using a ^241^Am gamma ray ~59.5 keV.

## Data Availability

The data supporting this study are available from the authors on reasonable request.
